# Integrative profiling of lactylation reveals prognostic biomarkers and an immunosuppressive niche in acute myeloid leukemia

**DOI:** 10.3389/fimmu.2026.1765979

**Published:** 2026-03-23

**Authors:** Zhibo Guo, Wenlei Zhang, Zengliang Gao, Qi Li, Dan Guo, Lijuan Yue, Yutong Liu, Xiaoting Ni, Shengjin Fan, Xin Hai

**Affiliations:** 1 Department of Pharmacy, First Affiliated Hospital of Harbin Medical University, Harbin, China; 2 Department of Hematology, First Affiliated Hospital of Harbin Medical University, Harbin, China; 3 National Health Commission (NHC) Key Laboratory of Cell Transplantation, Harbin, Heilongjiang, China

**Keywords:** acute myeloid leukemia, lactylation-related genes, leukemic microenvironment, machine learning algorithms, prognostic marker

## Abstract

**Background:**

The overall survival rate of acute myeloid leukemia (AML) remains less than 30%. Metabolic reprogramming of leukemia cells, such as the Warburg effect, enables them to adapt to the microenvironment and thereby develop. Elucidating the landscape of lactate regulation in AML helps clarify the pathogenesis from the perspective of metabolic reprogramming and identify possibilities for optimizing current treatment modalities.

**Methods:**

RNA and single-cell sequencing data for AML were obtained from the Gene Expression Omnibus (GEO) and The Cancer Genome Atlas (TCGA) databases. Seurat, limma package algorithm and Weighted gene coexpression network analysis (WGCNA) were conducted to identify candidate lactylation-related genes (LRGs). Enrichment analyses and protein-to-protein interactions were used to clarify the functions. Univariate COX regression and machine learning algorithms (LASSO-logistic, SVM-RFE and Boruta) narrowed the range of LRGs.The DALEX package employed four machine learning models for validation. CIBERSORT analyzed the relationship between immune cell infiltration and key LRGs, while single-gene GSEA was utilized to evaluate the functions of LRGs. We evaluated the associations between hub LRGs and AML using a two-sample Mendelian randomization (MR) analysis. Molecular docking was used to screen for feasible drugs targeting the hub genes. Western blotting was performed to assess pan-lactylation levels in AML cell lines. qRT–PCR and immunohistochemistry were performed to detect GZMB/LSP1 expression in AML patients.

**Results:**

Seven hub LRGs were identified in the AML groups: LSP1, MPO, GZMB, SPINK2, HLA-DRB1, HLA-DRA and POU2F2, of which GZMB and LSP1 passed MR test. The seven hub genes were enriched in immune and inflammatory pathways. GLM ultimately emerged as the optimal model validated by GEO datasets. Compared with healthy controls, Kasumi-1 cells exhibited elevated lactylation levels, with exogenous lactate treatment further increasing lactylation levels, whereas sodium oxamate administration had the opposite effect. Exogenous lactate treatment significantly upregulated the mRNA expression of GZMB and LSP1. (-)-Gallocatechin gallate and indomethacin bound well to GZMB, while benzo(a)pyrene and benzo(e)pyrene had good binding potential with LSP1.

**Conclusions:**

We established lactylation as a critical regulator of AML, and GZMB and LSP1 were identified as lactylation-related clinical modeling indicators, which provides a foundation for choosing prognostic and therapeutic strategies for AML.

## 1 Introduction

Acute myeloid leukemia (AML) is a lethal hematopoietic malignancy derived from abnormally proliferating stem cells, which are also called leukemic stem cells (LSCs) (1). Despite the development of chemotherapy, targeted therapy, immunotherapy and hematopoietic stem cell transplantation (HSCT), only 30% of AML patients can achieve five-year survival (2). The underlying mechanisms of AML development and progression, including gene mutations in LSCs, inordinate epigenetic regulation and the leukemic microenvironment (3, 4), have not been fully elucidated.

The bone marrow microenvironment (BMME) plays a key role in the regulation of hematopoiesis, but the molecular complexity and interactions among LSCs and the BMME remain incompletely understood (5). The BMME is composed of cells and molecules and then induces different fates of hematopoietic stem cells (HSCs). The main cellular components of the BMME include stromal cells, immune cells, endothelial cells, osteoblasts and adipocytes. Crosstalk between leukemic BMME and LSCs promotes the maintenance and progression of AML (6). Hypoxia and the accumulation of lactate are features of the malignant microenvironment (7, 8), and AML is no exception (9). Malignancies tend to use glycolysis, thus leading to the accumulation of lactate, which is also called the Warburg effect (10). Metabolic reprogramming leads to the accumulation of lactate, which not only promotes an acidic leukemic microenvironment but also acts as a modulator and accelerates leukemia processes (11, 12). A study found that lactate was the most significantly different metabolite between AML and normal human BMME (9). Lactate acid also promotes PD-1 + Tregs accumulation in the bone marrow with high tumor burden of acute myeloid leukemia (13).

The lactylation of proteins is a gradually elucidated posttranslational modification (PTM) that depends on the binding of lactate groups to lysine residues and thus impacts physiological processes (14, 15). Abnormal lactylation modulation occurs in both tumors and the microenvironment, resulting in the remodeling of immune cells to support immune evasion and tumor aggressiveness (16). Lactate accumulation and the modulation of lactylation have been confirmed to participate in AML progression and treatment failure (9, 17). Currently, there are exploratory studies on gene-related models (such as LMNA, RETN and HK1) regulating lactate metabolism in AML (18–20). However, no research has been conducted on the comprehensive regulatory network of genes directly influencing prognosis. Clarifying the roles of lactylation and its regulation in AML occurrence and progression is helpful for elucidating the mechanism of AML and providing a means of intervention.

Given the pivotal role of lactylation modulation in AML, identifying LRGs helps provide new insights into AML pathogenesis, potential risk stratification markers and treatment targets. In this study, we utilized multiple bioinformatic algorithms, machine learning techniques and molecular docking tools to analyze single-cell sequencing and transcriptome data from AML samples, with the goal of identifying LRGs closely associated with the MHC class II protein complex and immune modulation; of these LRGs, GZMB and LSP1 were validated to have causal relationships with AML through a Mendelian randomization analysis. Interestingly, common drugs such as indomethacin, dronabinol and dichlorvos might influence GZMB, thus inhibiting antitumor immune responses. Our findings provide novel insights into “lactate-lactylation-LRGs” molecular mechanisms in AML thus developing personalized therapeutic strategies.

## 2 Materials and methods

### 2.1 Ethics

Ethical approval for this study was provided by the Ethics Committee of the First Affiliated Hospital of Harbin Medical University.

### 2.2 Data acquisition

Single-cell data: The AML single-cell dataset (GSE116256) was downloaded from the NCBI GEO (http://www.ncbi.nlm.nih.gov/geo/) database. This dataset contains single-cell RNA sequencing (scRNA-seq) data from 16 AML patients and 4 healthy donors.

Transcriptome data: RNA-seq expression data from 173 AML patients from The Cancer Genome Atlas (TCGA) database (TCGA-LAML) and 70 normal bone marrow samples from the Genotype-Tissue Expression (GTEx) database were downloaded from the UCSC Xena database (https://xenabrowser.net/datapages/). TCGA data was used as the training set. The downloaded expression data were normalized by log2(tpm + 0.001). A total of 149 of these samples had prognostic information available (**Supplementary Table 1**). Moreover, datasets containing overall survival (OS) prognostic information were screened from the NCBI GEO database (http://www.ncbi.nlm.nih.gov/geo/) using “acute myeloid leukemia” as the keyword. The GSE71014, GSE12417, and GSE37642 datasets were selected for validation set, and samples with prognostic information were retained (**Supplementary Table 2**).

### 2.3 Cluster annotation and analysis of differentially expressed genes using single-cell data

Seurat (version 5.1.0, https://cran.r-project.org/web/packages/Seurat/index.html) was used to analyze the AML single-cell dataset GSE116256. Quality control (QC) was performed with the following criteria: nFeature_RNA > 200, nFeature_RNA < 3000 and mitochondrial genes < 20%. Principal component analysis (PCA) was used for dimensionality reduction, and Harmony (https://github.com/immunogenomics/harmony) was employed for batch correction. The “FindVariableFeatures” function identified the top 2000 variable genes. ElbowPlot was used to determine the optimal number of PCAs, and 30 principal components were selected by performing clustering analysis with a cutoff of 0.5. Cell type annotations were determined based on AML single-cell data using the “SingleR” package (21) (version 2.4.1, https://bioconductor.org/packages/release/bioc/html/SingleR.html).

Using the FindAllMarkers function from the Seurat package, we performed a differential expression analysis between different cell types and between the AML and normal groups. The criteria for identifying DEGs were |log2FC| > 0.5 and p.adj < 0.05, resulting in the identification of disease-related differentially expressed genes, and denoted as DEGs1.

### 2.4 DEG identification and functional annotation of transcriptome data

The limma package (22) (version 3.56.2; https://bioconductor.org/packages/release/bioc/html/limma.html) in R was used to screen for genes that were significantly differentially expressed between the patient group and the control group in TCGA dataset. The P value for a significant difference was calculated, and we evaluated it from two aspects: the fold change and the significance. The differential expression threshold was set as follows: p.adj < 0.05 and |log2FC| > 1. The genes that met these criteria were identified as differentially expressed genes, denoted as DEGs2.

### 2.5 Lactylation scores

A lactylated gene set consisting of 332 genes was obtained from the literature (23). The ssGSEA algorithm was applied, and the R package “GSVA” (24) (version: 1.50.0, http://bioconductor.org/packages/release/bioc/html/GSVA.html) was used to calculate the ssGSEA scores, thereby determining the lactylation scores for all the samples. The Wilcoxon test was used to examine the expression levels in the samples and the differences in scores between AML samples and control samples.

### 2.6 Weighted gene coexpression network analysis

WGCNA was performed on the top 2000 genes with the greatest absolute median differences in gene expression to screen for module genes related to the lactylation score. We used the R package “WGCNA” (25) (Version 1.72, https://cran.r-project.org/web/packages/WGCNA/) with the lactylation score as the phenotypic trait to screen for modules that were strongly correlated with the lactylation score. The intersection of these modules with the single-cell DEGs1 and the transcriptome DEGs2 yielded lactylation-related genes (LRGs).

### 2.7 Enrichment analysis and protein–protein interaction network

The “clusterProfiler” package (26) (version 3.14.3; http://bioconductor.org/packages/release/bioc/html/clusterProfiler.html) was used to perform a GO (Gene Ontology) analysis on key genes to determine the enrichment of BP (biological process), CC (cellular component), and MF (molecular function) terms and to conduct a KEGG (Kyoto Encyclopedia of Genes and Genomes) enrichment analysis, with a threshold screening p value < 0.05.

The STRING (version 12) database (27) (https://string-db.org) was used to predict and analyze the interactions among the proteins encoded by key genes. The PPI score was set to 0.4.

### 2.8 Univariate prognostic analysis

The “survminer” package (28) (version 0.4.9; https://cran.rstudio.com/web/packages/survminer/index.html) in R was used to perform univariate Cox regression analysis to calculate the relationships between the key genes and the prognosis of cancer, thereby yielding univariate prognostic genes.

### 2.9 Machine learning methods and clinical nomogram model

We employed multiple methods (29) for feature selection of univariate prognostic genes (1). The “e1071” package (version 1.6–8, https://cran.r-project.org/web/packages/e1071) was used to perform SVM-RFE feature selection with 10-fold cross-validation (https://github.com/johncolby/SVM-RFE). (2) The “glmnet” package provided by R (version 4.1-7; https://glmnet.stanford.edu) was used with 10-fold cross-validation, and the family was set to “binomial” for fitting. (3) The “Boruta” package provided by R (30) (version 8.0.0; https://gitlab.com/mbq/Boruta/) was used, with the maximum number of iterations for the algorithm set to 500. Finally, the feature genes selected by the different algorithms were compared, and the overlapping portion was retained as the lactylation feature genes.

### 2.10 Identification of hub genes as biomarkers

The expression of key genes in the training set (TCGA-LAML) was analyzed between the disease group and the control group to clarify the expression of lactylation signature genes in tumor samples and control samples. The ROC curves of each lactylation signature genes in the training set were constructed. Genes whose expression was significantly altered and whose AUC was > 0.7 were selected as biomarkers. The correlations between biomarkers were calculated, and a correlation heatmap was drawn. The KM curve of biomarkers was constructed.

### 2.11 AUCell scoring of single-cell data

The distribution of the expression of biomarkers identified in the single-cell data was visualized in UMAP plots. The PercentageFeatureSet function and AddModuleScore function were used to quantify the proportion of biomarker-related differentially expressed genes in each cellular subpopulation. The results of AddModuleScore were statistically analyzed separately for the disease and normal groups. Cells with significant differences and the highest expression levels were defined as key cells.

Using the biomarkers, we applied AUCell (version 1.24.0, https://bioconductor.org/packages/release/bioc/html/AUCell.html) to perform AUCell scoring on all tumor cells. The “AUCell_exploreThresholds” function was used to determine the AUC scoring threshold and identify active cells within the gene set. Based on the AUC scoring threshold, the cells were classified into high-activity and low-activity groups.

### 2.12 Cell communication analysis

CellChat (31) (version 1.6.1; https://github.com/sqjin/CellChat) was used to analyze intercellular communication among all annotated cells using the single-cell datasets. CellChat contains a database of human ligand–receptor interactions and can analyze the intercellular communication network based on single-cell sequencing data.

### 2.13 Construction of predictive models based on multiple machine learning methods

Diagnostic models were constructed using the biomarkers in validation set (GEO data mentioned above). The machine learning prediction models included the support vector machine (SVM), random forest (RF), generalized linear model (GLM), and extreme gradient boosting (XGB) models. The “DALEX” package (version 2.4.3, https://modeloriented.github.io/DALEX/) was used to interpret the four machine learning models and visualize the residual distribution and feature importance among the models.

Based on the normalized data from key cells (noncount data), the disease samples and control samples of key cells were randomly divided into 50 groups. After the mean of each group was taken, pseudobulk data were obtained for machine learning validation to determine the ability of the machine learning models to distinguish the disease and nondisease status of key cells. The AUCs of the diagnostic ROC curves from the four machine learning algorithms in the training cohort and single-cell pseudobulk data cohort were evaluated and visualized using the “pROC” R package.

### 2.14 GSEA of cellular components of the BMME

The CIBERSORT algorithm (32) was used to evaluate the levels of 22 immune cells in all the samples in the training cohort as a method to understand the BMME in AML. The difference in the abundance of immune cell infiltration in the training cohort was analyzed. Spearman’s correlation analysis was performed to analyze the correlations between differentially abundant immune cells and biomarkers in the training cohort to explore the correlation between the BMME and biomarkers in AML.

KEGG and HALLMARK were selected as the reference gene set for the MSigDB database to investigate the molecular pathways related to biomarkers. Single genes were sorted according to their correlations with other genes. The GSEA software R package “ClusterProfiler” was used for the enrichment analysis.

### 2.15 Mendelian randomization analysis

We utilized seven sets of expression quantitative trait loci (eQTLs) data as exposures to investigate the causal relationship between genetic variants and AML. All eQTL data were obtained from the IEU Open GWAS Project (https://opengwas.io/datasets). For each eQTL dataset, single nucleotide polymorphisms (SNPs) exhibiting significant associations (SNP p value < 5x10^-8^) were selected as instrumental variables (IVs). Linkage disequilibrium (LD) was removed by applying thresholds of r² < 0.001 and kb = 10,000, and weak instruments were excluded based on the F statistic. These SNPs were subsequently extracted from AML GWAS summary statistics and harmonized using the harmonize function from the TwoSampleMR R package.

The AML GWAS summary statistics were sourced from the European Bioinformatics Institute (EMBL-EBI) GWAS Catalog (https://www.ebi.ac.uk/gwas/), with the dataset identifier GCST90043912. This dataset includes 220 AML patients and 456,128 controls.

### 2.16 Identification of candidate small-molecule drugs

The DGIdb database (33) (Drug–Gene Interaction database, http://dgidb.org/) was used to browse, search, and filter information on drug–gene interactions and druggable genes, predicting all drug–gene relationship pairs related to the biomarkers. Using the PubChem database (34) (https://pubchem.ncbi.nlm.nih.gov/), the 3D structures of the higher-ranked drug components were subsequently identified, and the 3D structures of the target proteins were identified in the PDB database (https://www.rcsb.org/). The PDB data for the corresponding complexes were downloaded, and PyMOL (35) (version 2.4.0, https://www.lfd.uci.edu/~gohlke/pythonlibs/) was used to remove the water molecules from the complexes and remove all other molecules, after which only the PDB data for the target proteins were retained. Then, AutoDock (36, 37) (version 4.2.6, https://vina.scripps.edu/) was used for molecular docking of active drugs and target proteins, and LigPlot+ (38) (v.2.2, https://www.ebi.ac.uk/thornton-srv/software/LigPlus/) software was used to visualize the docking results.

### 2.17 Cell culture and drug preparation

Peripheral blood mononuclear cells (PBMCs) were isolated from healthy donors using Ficoll–Paque density gradient centrifugation (GE Healthcare, Chicago, IL, USA). The human AML cell lines Kasumi-1 and THP-1 were obtained from the ATCC (Manassas, VA, USA; CRL-2724 and TIB-202, respectively). All cells were cultured in RPMI-1640 medium (Gibco, Thermo Fisher Scientific, Waltham, MA, USA) supplemented with 10% fetal bovine serum (FBS, Gibco), 100 U/mL penicillin/streptomycin (Gibco), and 2 mM L-glutamine (Gibco) at 37 °C in a 5% CO_2_ humidified incubator (Thermo Fisher Scientific).

### 2.18 Drug reconstitution

Sodium oxamate and sodium lactate (MCE; Monmouth Junction, NJ, USA) were dissolved in sterile phosphate-buffered saline (PBS) to prepare 20 mM stock solutions. Working concentrations (0.5–20 mM) were prepared fresh daily and filter-sterilized (0.22 μm, Millipore, MA, USA).

### 2.19 Cell viability assay (CCK-8)

Cell proliferation was evaluated using a Cell Counting Kit-8 (CCK-8; Dojindo Molecular Technologies, Kumamoto, Japan). Briefly, 5×10^3^ cells/well were seeded in 96-well plates and treated with gradient concentrations of the test compounds. After 24 h of incubation, 10 μL of CCK-8 reagent was added, and the samples were incubated for 1–4 h at 37 °C. The absorbance at 450 nm was measured using a microplate reader (BioTek Synergy H1, Winooski, VT, USA). Cell viability was calculated as follows:


% Viability = [OD(test) − OD(blank)]/[OD(control) − OD(blank)] × 100

The data are presented as the means ± SDs of three independent experiments (n=3).

### 2.20 RNA extraction and quantitative RT–PCR

Total RNA was extracted using TRIzol reagent (Invitrogen, CA, USA) according to the manufacturer’s protocol. Exosomal RNA was isolated using the Total Exosome RNA & Protein Isolation Kit (Invitrogen), as previously described. RNA integrity was verified using a Nanodrop ND-1000 (A260/A280 ≥1.8; Thermo Fisher). First-strand cDNA was synthesized using BeyoRT II M-MLV Reverse Transcriptase (Beyotime, CN) for mRNA or the miRcute Plus miRNA First-Strand cDNA Synthesis Kit (Tiangen, Beijing, CN) for miRNA, according to the suppliers’ instructions. Quantitative PCR was performed on an Applied Biosystems 7500 Real-Time PCR System using SYBR^®^ Premix Ex Taq™ II (Takara, JP). The primer sequences are listed in **Table 1**. The cycling conditions were 95 °C for 30 s (initial denaturation), followed by 40 cycles of 95 °C for 5 s and 60 °C for 30 s. β-Actin served as an endogenous control. Relative expression levels were calculated using the 2^-ΔΔCt^ method.

**Table 1:** Primers for circRNAs, miRNAs and mRNAs measured by qRT–PCR.

RNA	Sequence
GZMB-F	CGACAGTACCATTGAGTTGTGCG
GZMB-R	TTCGTCCATAGGAGACAATGCCC
LSP1-F	TGAAGTCCACCTGGAGGAGTTG
LSP1-R	TGGGCTGCTGACATTTCTGGTG
β-actin-F	GGGAAATCGTGCGTGACATT
β-actin-R	GGAACCGCTCATTGCCAAT

### 2.21 Western blot analysis

Proteins were extracted using RIPA lysis buffer (Beyotime) supplemented with protease inhibitors (Roche, Basel, CH). The total protein concentration was quantified using a BCA assay (Thermo Fisher). Equal amounts of proteins (20 μg) were separated on 10% SDS–PAGE gels and transferred to PVDF membranes (Millipore) using a Trans-Blot Turbo system (Bio-Rad). The membranes were blocked with 5% nonfat milk in TBS-T for 1 h at room temperature. Primary antibodies against L-lactyl lysine rabbit mAb (1:1000, PTM-1401, PTM Bio, China) and β-actin (1:5000, Sigma, MO, USA) were incubated with the membranes overnight at 4 °C. Horseradish peroxidase-conjugated secondary antibodies (1:5000; Jackson ImmunoResearch, PA, USA) were applied and incubated for 1 h. The signals were detected using enhanced chemiluminescence (ECL; Thermo Fisher) and quantified by densitometry (Image Lab 6.0; Bio-Rad, CA, USA).

### 2.22 Immunohistochemistry

Bone marrow biopsies from AML patients and healthy controls were fixed with 10% neutral-buffered formalin (Leica Biosystems, Nussloch, DE) for 24–48 h, embedded in paraffin, and sectioned at a 4 μm thickness. Deparaffinized sections underwent antigen retrieval using citrate buffer (pH 6.0) in a pressure cooker (121 °C, 20 min). Endogenous peroxidase activity was blocked with 3% H_2_O_2_ for 10 min. Primary antibodies against GZMB (1:200; Abcam, UK) and LSP1 (1:150; Santa Cruz Biotechnology, TX, USA) were applied for 1 h at room temperature. Signals were amplified using the EnVision+ System (Dako, Glostrup, DK) with diaminobenzidine (DAB) as the chromogen. The sections were counterstained with hematoxylin, dehydrated, and mounted.

### 2.23 Statistical analysis

The data are presented as the means ± SDs. GraphPad Prism 8.0 (GraphPad Software, La Jolla, CA, USA) was used to perform the analyses. Student’s t tests were used to estimate differences between two groups. One-way analysis of variance (ANOVA) was used to compare three or more groups. Pearson’s correlation analysis was conducted to analyze the associations between the hub genes and immune cells. A p value<0.05 was considered to indicate statistical significance.

For the MR analysis, five methods were employed to assess the causal effect of exposures on the outcome: inverse variance weighted (IVW), MR–Egger, weighted median, simple mode, and weighted mode. Among these, the IVW method served as the primary analytical approach, while the others were used for supplementary analyses. Pleiotropy was assessed using the MR–Egger intercept and the MR Pleiotropy Residual Sum and Outlier (MR-PRESSO) test. A P value > 0.05 indicated the absence of pleiotropic effects. Heterogeneity was evaluated using Cochran’s Q statistic derived from both MR–Egger and IVW analyses, with a P value > 0.05 suggesting no significant heterogeneity. The causal effects are reported as odds ratios (ORs) along with their 95% confidence intervals (CIs). All the statistical analyses were conducted in R, and a P value < 0.05 was considered to indicate statistical significance.

## 3 Results

### 3.1 Cellular heterogeneity in AML based on a single-cell atlas

**Figure 1:**
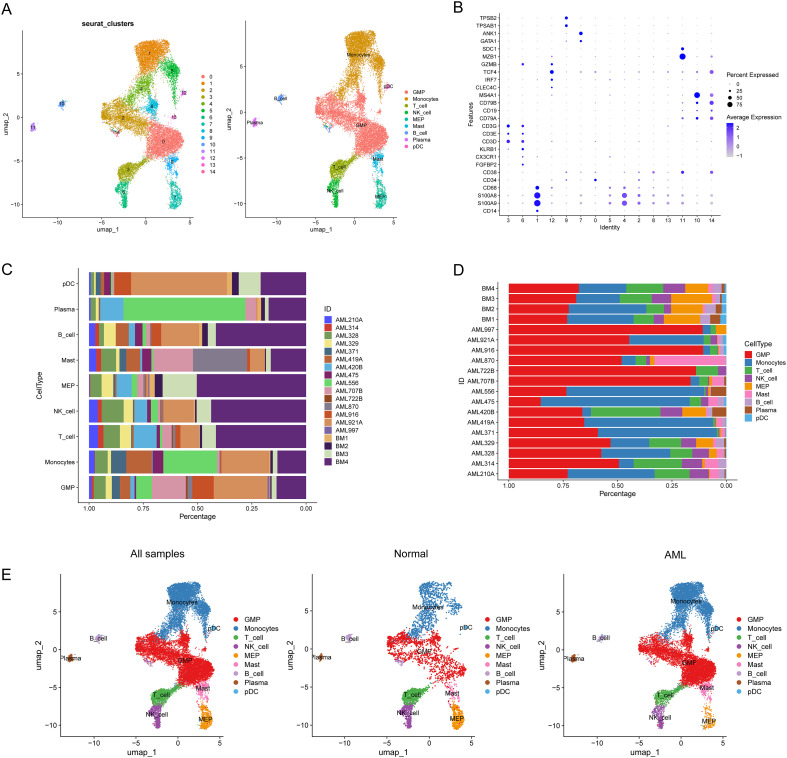
AML single-cell atlas. **(A)** Fifteen cell clusters were obtained, resulting in the identification of nine cell types: granulocyte–monocyte progenitor cells (GMPs), monocytes, T cells, NK cells, megakaryocyte–erythroid progenitor cells (MEPs), mast cells, B cells, plasma cells, and plasmacytoid dendritic cells (pDCs). Left: UMAP map classified by sample type; right: UMAP map classified by cell markers. **(B)** Dot plots of standardized markers for nine cell types. **(C, D)** The percentage clusters are based on cell types and patient IDs. **(E)** UMAP plots of all cells colored according to normal and AML samples, indicating the hematopoietic cell components of the bone marrow microenvironment clustered by cell type.

We conducted a UMAP analysis of the AML single-cell dataset (GSE116256) to elucidate the cellular composition in the leukemic microenvironment and the functional characteristics of AML. Fifteen cell clusters were obtained, and the cell clusters were annotated using recognized cell markers Finally, merge them into nine cell types based on the expression of standardized markers(**Figures 1A, B**): granulocyte–monocyte progenitor (GMP) cells, monocytes, T cells, NK cells, megakaryocyte–erythroid progenitor (MEP) cells, mast cells, B cells, plasma cells, and plasmacytoid dendritic cells (pDCs). The GMP cells exhibited high expression of CD34 and CD38. MEP cells expressed GATA1 and ANK1. Monocytes specifically expressed high levels of CD14, S100A9, S100A8 and CD68. NK cells were identified based on their expression of specific markers, including FGFBP2, CX3CR1 and KLRB1. Mast cells were identified by TPSAB1 and TPSB2 expression. pDCs expressed CLEC4C, IRF7, TCF4 and GZMB. Additionally, three major lineage populations were recognized based on the gene expression patterns: T cells (CD3D, CD3E, and CD3G), B cells (CD19, CD79A, CD79B and MS4A1), and plasma cells (MZB1 and SDC1).

Significant differences in the cellular composition were observed between AML patients and healthy donors (ID: BM1-4). The percentage of cells was visualized according to cell type and sample ID (**Figures 1C, D**), which indicated that AML was highly heterogeneous and characterized by patient-specific expression (**Figure 1E**). In most AML samples, the number of GMP cells was significantly greater than that in healthy donors, which indicated that the numbers of LSCs and blasts were relatively high in AML patients.

### 3.2 Identification of DEGs based on single-cell and transcriptome data

**Figure 2:**
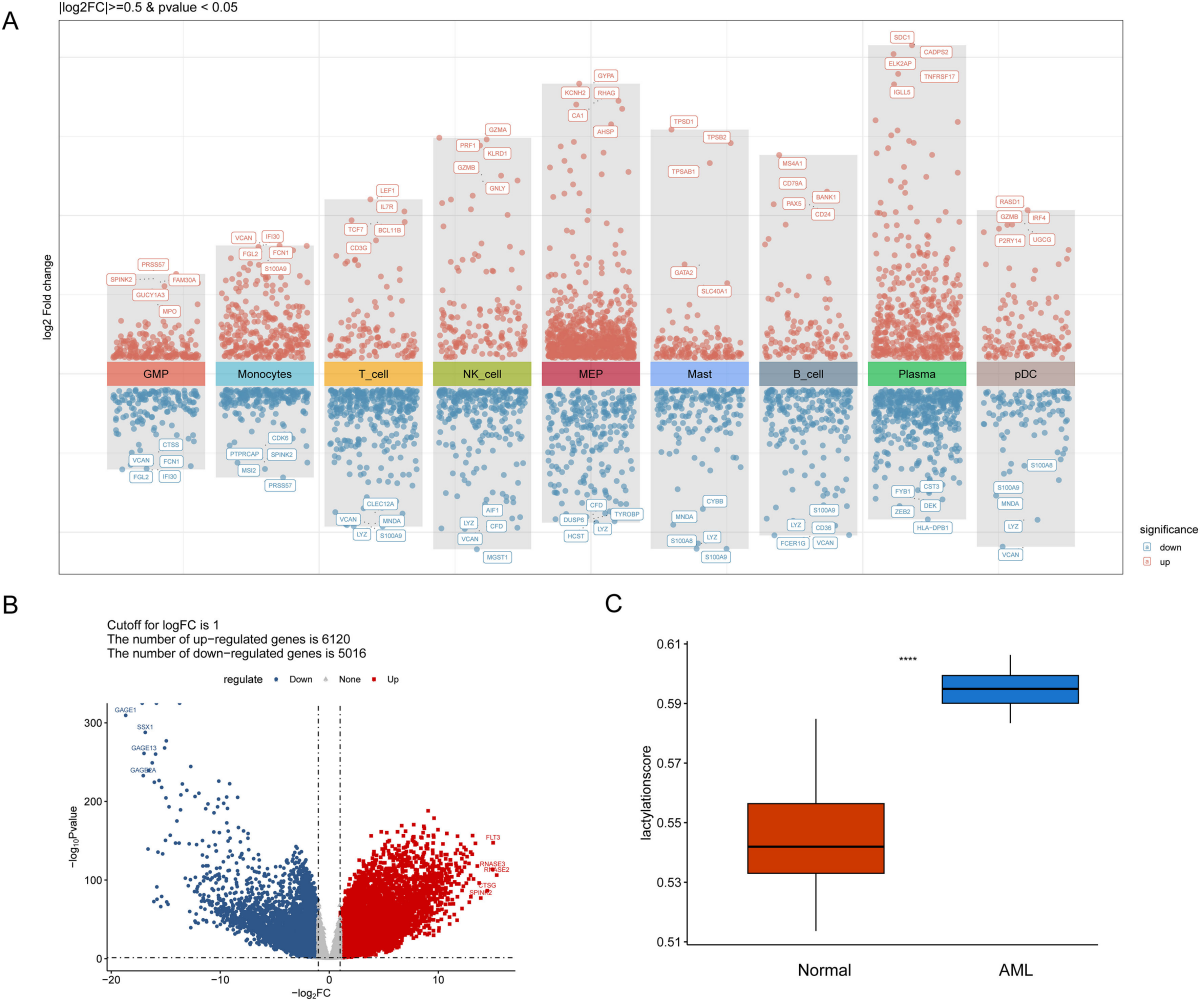
Identification of DEGs based on single-cell and transcriptome data. **(A)** A total of 3984 differentially expressed genes were identified based on single-cell data and referred to as DEGs1 (cutoff: |log2FC|>=0.5, p value<0.05). **(B)** Volcano plot of the 6120 upregulated genes and 5016 downregulated genes identified in AML samples compared with normal samples and referred to as DEGs2 (cutoff: |log2FC|>=1, p value<0.05). **(C)** Box plot of lactylation scores for AML and normal samples. ****p value < 0.0001.

To identify the key targets, we first conducted an analysis of disease-related DEGs. A differential expression analysis was performed for the AML vs. normal groups based on the single-cell and TCGA LAML datasets, resulting in the identification of 3984 differentially expressed genes, called DEGs1 (**Figure 2A**), and 11,136 differentially expressed genes (**Figure 2B**), called DEGs2 (**Supplementary Tables 3**, **4**), respectively.

### 3.3 Identification of key module genes associated with lactylation in AML

To further explore the role of lactylation in AML, we conducted lactation scoring and then identifying key lactation-related module genes. In the ssGSEA algorithm, the “GSVA” package was used to calculate the lactylation scores for all samples in the training cohort, and the differences in scores between disease samples and control samples were compared. The lactylation score of AML was relatively high (**Figure 2C**).

**Figure 3:**
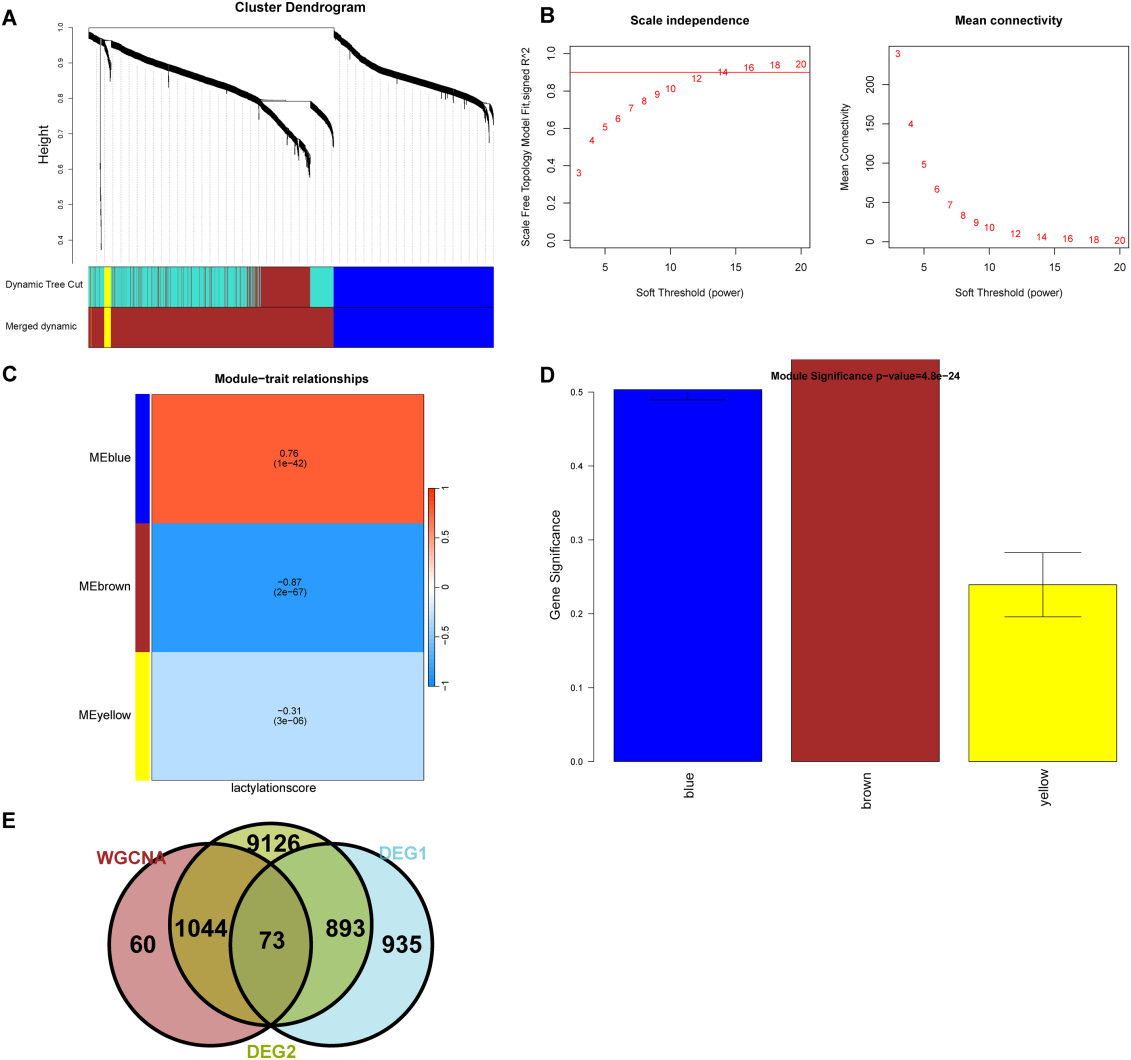
WGCNA and identification of key module genes. **(A-D)** WGCNA revealed that the most relevant module associated with AML, the MEbrown module, positively correlated with AML. **(E)** Seventy-three key LRGs associated with AML.

WGCNA was performed in the top 2000 genes with absolute median expression in the training set to further identify key module genes associated with AML. Sample clustering indicated the absence of outliers in the analysis. A soft threshold power of 14 was selected as optimal, achieving a signed R² of 0.85, which supported a scale-free network topology as the average connectivity approached zero (**Figures 3A, B**). Using a dynamic tree-cutting algorithm with subsequent merging of similar modules, 3 distinct modules were identified (**Figure 3C**). Among these modules, MEbrown was significantly correlated with AML (|cor| > 0.6, p value<0.05) (**Figure 3D**). Modules highly correlated with lactylation scores were intersected with differentially expressed genes from the single-cell and transcriptome to obtain the final differentially expressed lactylation-related genes. A total of 73 genes were identified in AML by overlapping the key module genes of DEGs1, DEGs2 and WGCNA and then referred to as LRGs (**Figure 3E**, **Supplementary Table 5**).

### 3.4 Functional enrichment analysis of key module genes

**Figure 4:**
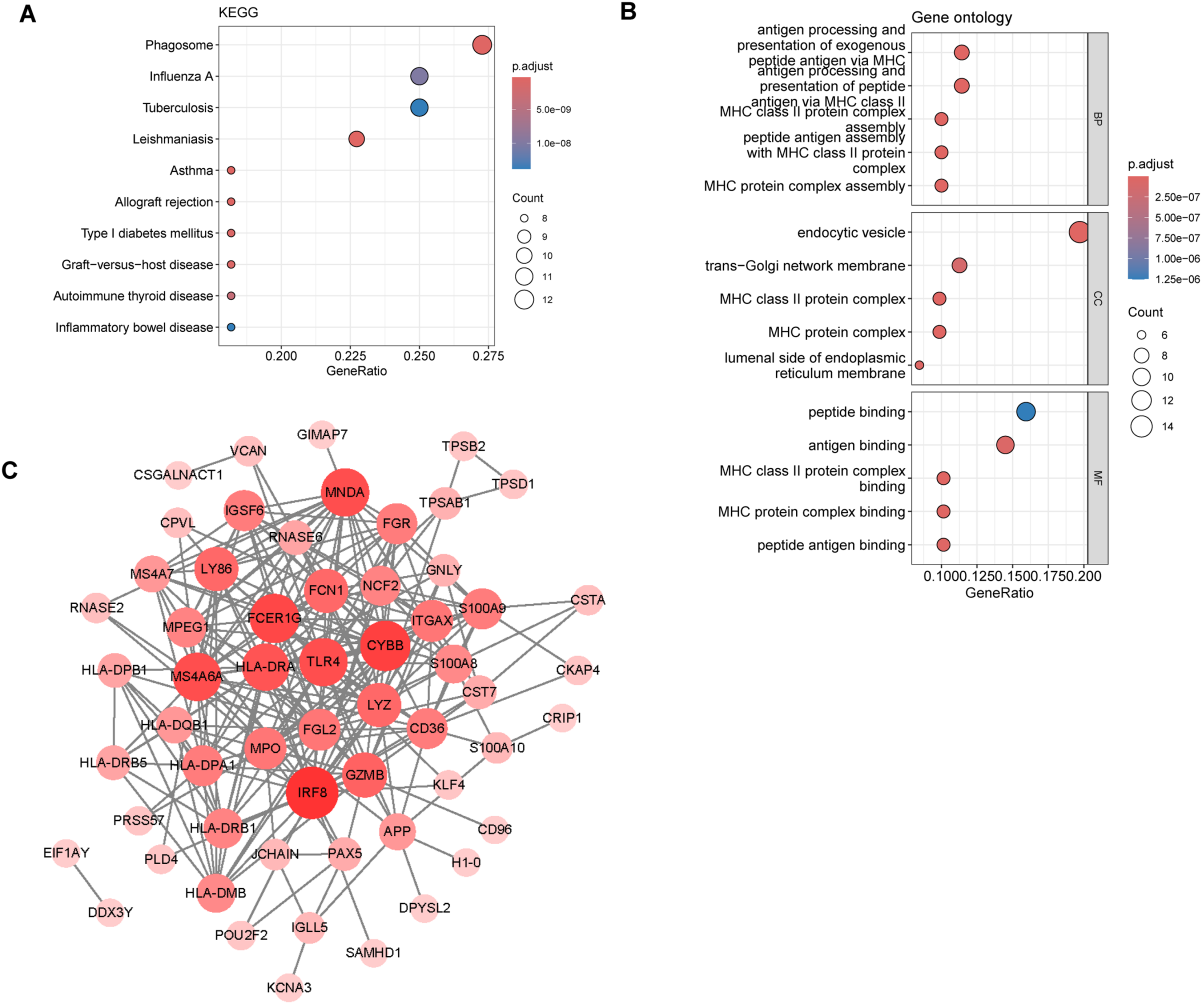
Functional enrichment analysis of key LRGs. **(A)** KEGG pathway enrichment analysis of key LRGs in AML. The top 10 enriched pathways are displayed. The nodes are the enriched genes, and the node color corresponds to the up- or downregulation of these genes. **(B)** Go, Gene Ontology enrichment analysis of key LRGs in AML. The top 10 progresses are displayed. **(C)** PPI, Protein–protein interaction network of key LRGs. The size of the node and the depth of the color represent the degree value.

KEGG and GO functional enrichment analyses revealed 73 LRGs, and a total of 27 KEGG pathways (**Figure 4A**) and 172 GO BP terms (**Figure 4B**) were coenriched. The KEGG pathway analysis revealed enrichment in pathways related to immunity and inflammation. Antigen presentation and other immune responses mediated by MHC class II molecules might play key roles in the regulation of lactylation in AML. The GO analysis indicated that the DEGs were associated mainly with “MHC class II protein complex”, “MHC class II protein complex binding” and “MHC class II protein complex assembly”. The PPI network is presented in **Figure 4C**, with 58 genes and 247 interactions. The top 10 genes with the most interactions were IRF8, CYBB, FCER1G, MNDA, TLR4, MS4A6A, HLA-DRA, GZMB, LY86 and CN1.

### 3.5 Machine learning to screen and identify lactylation signature genes in AML

**Figure 5:**
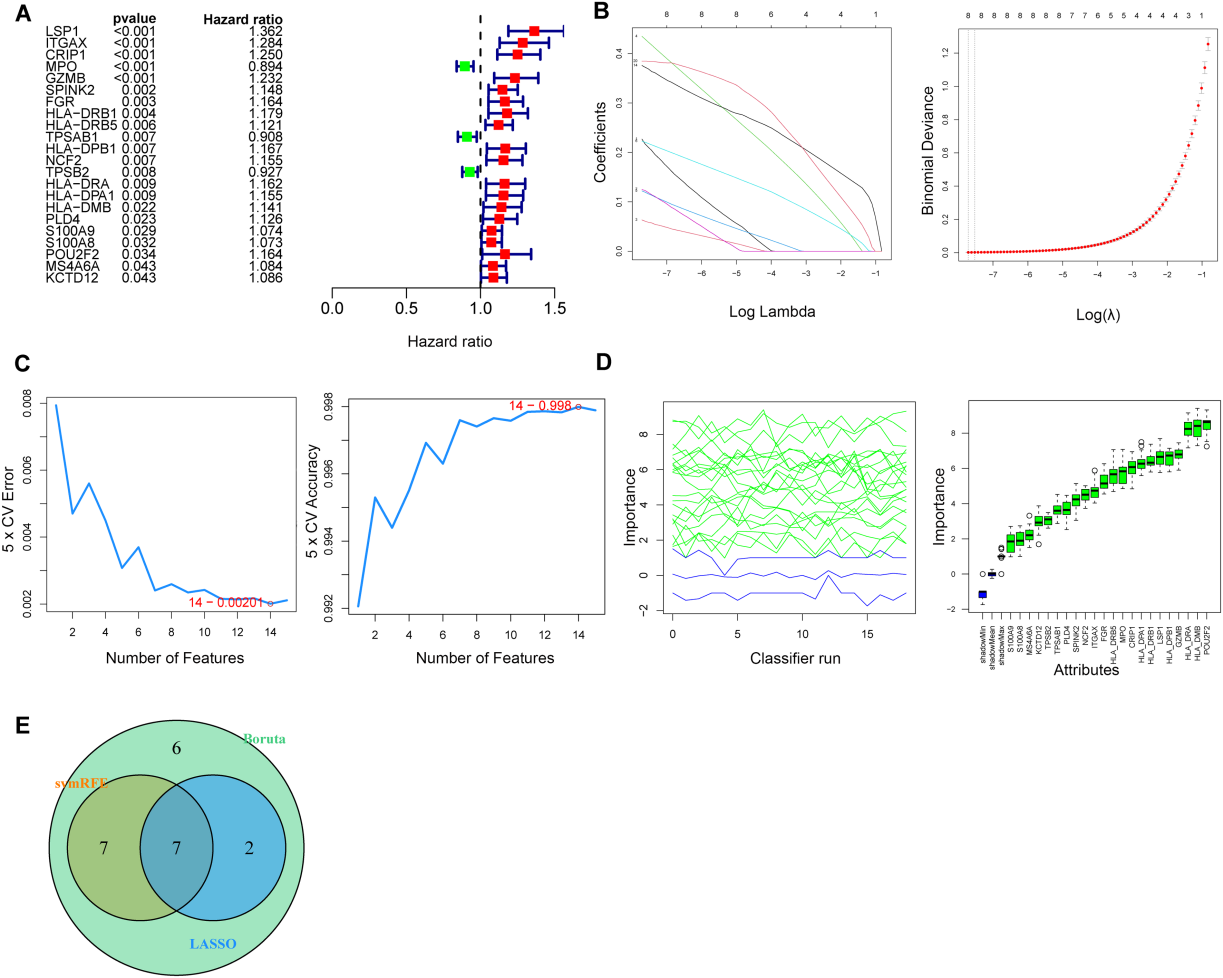
Screening characteristic LRGs in AML using machine learning. **(A)** Cox regression analysis was used to identify 22 significantly differentially expressed prognosis-related genes. **(B-D)** Screening characteristic LRGs in AML based on the LASSO-logistic **(B)**, SVM-RFE **(C)**, and Boruta **(D)** algorithms. **(E)**Venn diagram showing the intersections of the features screened using the aforementioned method, with 7 genes identified as lactylation signature genes.

Univariate Cox regression analysis was used to identify significantly differentially expressed prognosis-related genes as hub genes, resulting in the identification of a total of 22 genes (**Figure 5A**, **Supplementary Table 6**). The LASSO-logistic, SVM-RFE, and Boruta algorithms (**Figures 5B–D**) were used to obtain the respective features of the 22 single-factor prognostic genes. A Venn diagram was subsequently generated to extract the intersections of the features screened using the aforementioned methods, resulting in the identification of 7 genes as subsequent lactylation signature genes, including LSP1, MPO, GZMB, SPINK2, HLA-DRB1, HLA-DRA and POU2F2 (**Figure 5E**).

**Figure 6:**
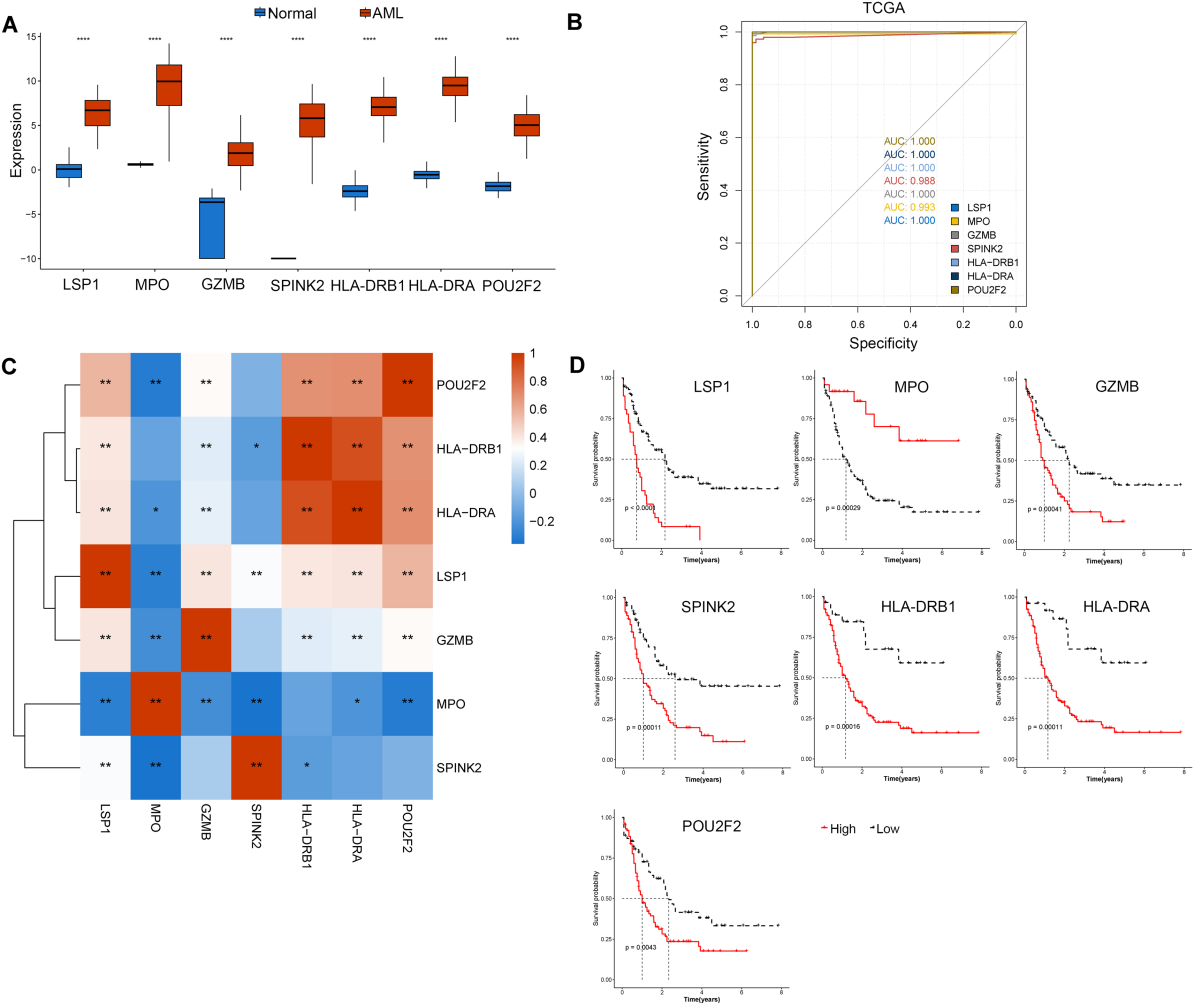
Identification of characteristic LRGs related to the AML prognosis. **(A)** Box diagram showing differentially expressed genes in AML and normal samples. **(B)** The ROC curves of characteristic LRGs in the training sets. **(C)** Correlation heatmap of characteristic LRGs. **(D)** KM curve based on the GEO dataset. *p value < 0.05, **p value < 0.01, ****p value < 0.0001.

Based on transcriptome data, the expression of key genes in the training set was compared between the disease and control groups to clarify the expression of lactylation signature genes in disease samples and control samples, as shown in **Figure 6A**. The ROC curves of the lactylation signature genes in the training set were calculated, and the AUCs of all the genes except for SPINK2 were greater than 0.99, indicating high accuracy and robustness (**Figure 6B**). The correlations between biomarkers were calculated, and a correlation heatmap is shown in **Figure 6C**. The KM curve of biomarkers was constructed (**Figure 6D**). The above results suggest that the prognostic model based on the 7 genes has good predictability and was further verified in the validation set.

### 3.6 Analysis of lactylation signature genes in the BMME

**Figure 7:**
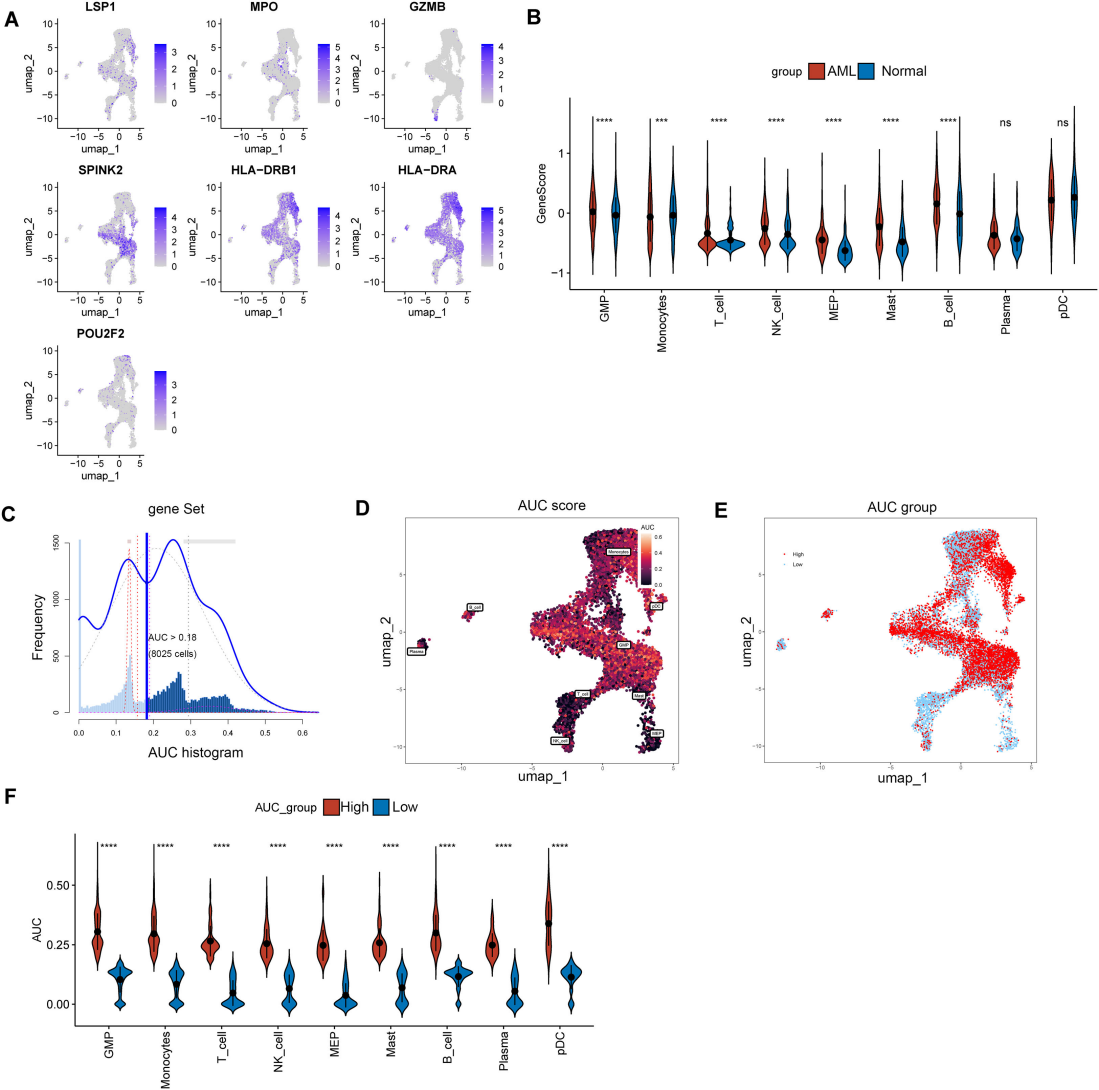
Analysis of lactylation signature genes in the BMME. **(A)** UMAP scatter plot of lactylation signature genes in the BMME. **(B)** Box plot of differences in lactylation signature gene scores across cells. **(C-E)** AUC scores. In order, an AUC score distribution map **(C)** is shown, and the AUC score **(D)** and high- and low-activity groups **(E)** are shown. **(F)** Box plot of the differences in each cell type between the high and low AML groups. ***p value < 0.001, ****p value < 0.0001, ns, not significant.

Based on the single-cell data, the distribution of the expression of biomarkers was visualized in UMAP plots (**Figure 7A**). The proportion of biomarker-related differentially expressed genes in each cell subpopulation was quantified, and the differences between the AML and normal groups were compared. The GMP cells showing significant differences in expression and the highest expression were defined as key cells (**Figure 7B**).

By performing an AUCell analysis, we calculated the AUC for each cell. Based on the AUC threshold of 0.18 determined by the AUCell_exploreThresholds function (**Figure 7C**), all cells were divided into a high-activity scoring group and a low-activity scoring group (**Figures 7D, E**). A comparison of the composition of various cell types between the high- and low-scoring groups in the disease group revealed significant differences in the expression of the key gene GMP (**Figure 7F**).

### 3.7 Influence of lactylation signature genes on BMME cell components in AML

**Figure 8:**
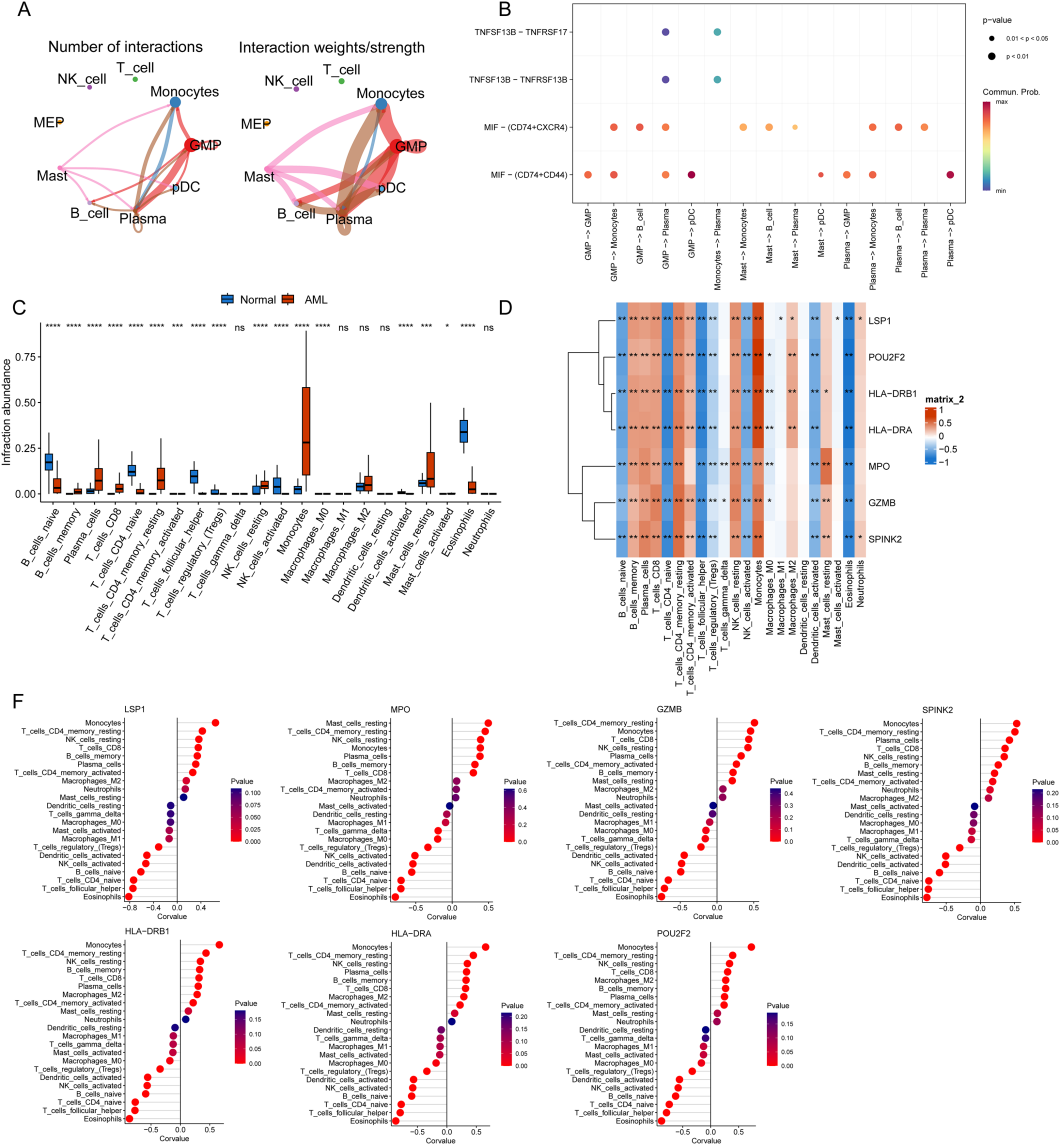
Analysis of lactylation signature genes in cellular components of the BMME. **(A)** Network diagram of the cellular communication of each subpopulation; thicker lines indicate more numbers and stronger interactions. **(B)** Bubble diagram of receptor–ligand pairs in cells. Each column has two cell subsets, and each behavior has a pair of receptor and ligand names. The color represents the average expression level of these two genes in the two subsets. The redder the expression is, the higher the expression is, and the bubble size represents the -log10 value of the p value. **(C)** The CIBERSORT algorithm was used to determine differences in immune cell types and the results are shown in a box plot. **(D)** Heatmap of gene and immune cell correlations. **(E)** Lollipop chart of the correlation between lactylation signature genes and cellular components of the BMME. *p value < 0.05, **p value < 0.01, ***p value < 0.001, ****p value < 0.0001, ns, not significant.

Considering the link between AML pathophysiology and the BMME, we further investigated cellular communication and immune cell distributions in AML using single-cell sequence data. CellChat was used to construct interaction network diagrams between each cell type and the other cell types. A cell communication analysis could help us understand the interactions between cells, parse the intercellular communication network, and reveal the interactions of various cells during development. As shown in the diagrams, only the interactions among GMPs, monocytes, plasma cells, B cells, and pDCs were relatively strong (**Figure 8A**). In the single-cell analysis, ligand–receptor interactions are key to studying intercellular communication. CellChat calculates the communication probability at the signaling pathway level by summarizing the communication probabilities for all ligand–receptor interactions associated with each signaling pathway (**Figure 8B**).

The CIBERSORT algorithm and ssGSEA were used to analyze the immune cell composition (**Figure 8C**). Notably, the abundances of 17 immune cell types, including naïve and memory B cells, plasma cells, CD8^+^ T cells, naïve CD4^+^ T cells, resting and activated memory CD4^+^ T cells, follicular helper T cells, regulatory T cells, resting and activated NK cells, monocytes, M0 macrophages, activated dendritic cells, and resting and activated eosinophils, differed significantly between the AML samples (**Figures 8D, E**). Further analysis revealed strong correlations between key biomarkers (LSP1, MPO, GZMB, SPINK2, HLA-DRB1, HLA-DRA, and POU2F2) and specific immune cells: in addition to monocytes, which might be responsible for AML, memory T cells were strongly positively correlated, whereas eosinophils, follicular T cells and naïve CD4^+^ T cells were significantly negatively correlated (**Figure 8F**).

### 3.8 Significance of the hub genes in the prediction models and enriched pathways

**Figure 9:**
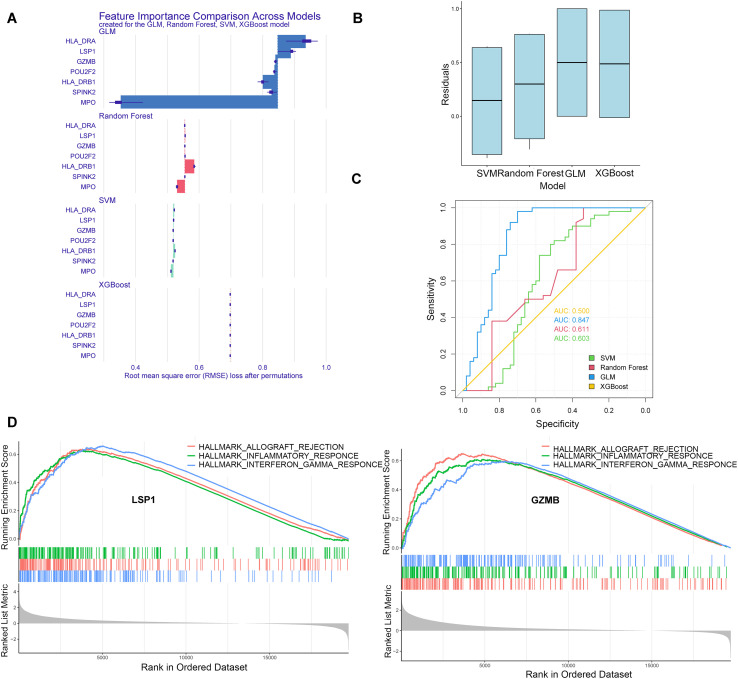
Prediction models based on 4 machine learning models and enriched pathways. **(A, B)** Four machine learning models were used to construct a diagnostic model based on the biomarkers and to visualize the residual distribution and feature importance between the models. **(C)** ROC curves of the training cohort and the single-cell bulk data cohort, and GLM was confirmed as the best machine learning model. **(D)** Samples were divided into high and low groups based on the median expression of the seven biomarkers and further analyzed using GSEA (LSP1 and GZMB were listed, the others were shown in **Supplementary Figure 1**).

Four machine learning models were used to verify diagnostic models based on the biomarkers in validation set, and the residual distributions and feature importance among the models were visualized (**Figure 9A**). The AUC values of the diagnostic ROC curves in the training cohort and the single-cell pseudobulk data cohort are shown in **Figures 9B, C**. Ultimately, GLM was identified as the best-performing machine learning model.

[KEGG, HALLMARK] was selected as the reference gene set, and the single genes were ranked according to their correlation with other genes using MSigDB to study the molecular pathways related to the biomarkers in a targeted manner. We divided the samples into high and low groups based on the median expression of the seven biomarkers according to the methods described above. A differential expression analysis was then performed, followed by GSEA (KEGG). The significance threshold was set at P < 0.05. Using the function “gseaplot2”, GSEA enrichment plots were generated for the top three pathways ranked by the NES (**Figure 9D**). GSEA for HALLMARKS indicated that MPO and SPINK2 were related to the epithelial–mesenchymal transition; LSP1 and GZMB were related to inflammation and IFN-gamma pathways; POU2F2 was related to cytokines and inflammation; LSP1, HLA-DRA and GZMB were related to allograft rejection; and HLA-DRB1 was related to immunity and antigen presentation (**Figure 9D**, **Supplementary Figure 1**). The results of the GSEA for KEGG pathways were similar to those of HALLMARKS and are shown in **Supplementary Figure 2**.

### 3.9 MR analysis revealed positive causal associations among GZMB expression, LSP1 expression and the risk of AML

To further narrow down the range of key LRGs, we conducted MR analysis on 7 genes to explore the causal relationship with AML. The results of the MR analysis indicated a positive causal association between GZMB and the risk of AML (IVW: P< 0.05; OR = 1.739, 95% CI: 1.214–2.490; all SNPs with P< 5x10^-8^) (**Supplementary Figure 3A**, **Table 2**). The number of SNPs was insufficient to perform an MR-PRESSO analysis. Nevertheless, all other sensitivity analyses suggested that the above findings were free from significant heterogeneity and pleiotropy. The leave-one-out analysis demonstrated that the MR results were not driven by any single SNP (**Supplementary Figure 3B**).

**Table 2:** The causal association of eQTLs with AML in the MR analysis.

Exposure	IVW		MR egger		Weighted median		Simple mode		Weighted mode	
	OR (95% CI)	*P*	OR (95% CI)	*P*	OR (95% CI)	*P*	OR (95% CI)	*P*	OR (95% CI)	*P*
GZMB	1.739 (1.214–2.490)	2.52E-03	1.760 (0.949–3.263)	0.324	1.728 (1.217–2.454)	2.2E -03	1.577 (0.869–2.863)	0.273	1.751 (1.199–2.557)	0.101
LSP1	3.951 (1.839–8.489)	4.29E-04	11.406 (1.816–71.650)	0.234	4.214 (1.821–9.752)	7.7E -04	3.024 (0.700–13.060)	0.276	2.519 (1.373–4.619)	7.25E -02
MPO	1.042 (0.702–1.548)	0.837	0.807 (0.481–1.354)	0.448	0.945 (0.623–1.433)	0.788	2.299 (0.697–7.581)	0.213	0.921 (0.599–1.417)	0.719
SPINK2	0.866 (0.436–1.720)	0.682	1.918 (0.726–5.068)	0.319	0.925 (0.502–1.704)	0.802	0.890 (0.381–2.081)	0.806	0.874 (0.463–1.653)	0.707
HLA-DRB1	1.151 (0.872–1.520)	0.320	1.321 (0.433–4.027)	0.711	1.163 (0.878–1.541)	0.293	1.400 (0.754–2.601)	0.398	1.157 (0.869–1.541)	0.423
HLA-DRA	0.976 (0.344–2.768)	0.964	0.404 (0.009–17.481)	0.720	0.875 (0.273-2.804)	0.823	0.850 (0.222–3.248)	0.834	0.863 (0.224–3.320)	0.850
POU2F2	1.228 (0.132–11.396)	0.856	3.72E05 (6.65E-04– 2.08E14)	0.430	2.402 (0.181–31.819)	0.506	3.313 (0.095–115.708)	0.577	3.837 (0.126–122.201)	0.526

Additionally, for LSP1, only three SNPs were identified from the eQTL data when a SNP P value threshold of < 5x10^-6^ was used. All three SNPs exhibited strong associations with exposure, as confirmed by F statistics (F>10). A subsequent MR analysis suggested a positive causal relationship between LSP1 and the AML risk (IVW: P< 0.05; OR = 3.951, 95% CI: 1.839–8.489; weighted median: *P* < 0.05; OR = 4.214, 95% CI: 1.821–9.752) (**Supplementary Figure 3C**, **Table 2**). Sensitivity analyses indicated no significant heterogeneity or pleiotropy in these results. The leave-one-out analysis further confirmed that the MR findings were robust and not influenced by any individual SNP (**Supplementary Figure 3D**).

For POU2F2, only three SNPs were selected from the eQTL data using a SNP P value threshold of < 5×10^-5^. Although these SNPs were confirmed to be strongly associated with exposure (F>10), subsequent MR analyses did not identify any significant causal relationships, and the remaining four genes did not demonstrate significant causal associations (**Table 2**, **Supplementary Table 7**).

### 3.10 Influence of lactate on the expression of GZMB and LSP1 in AML patients

**Figure 10:**
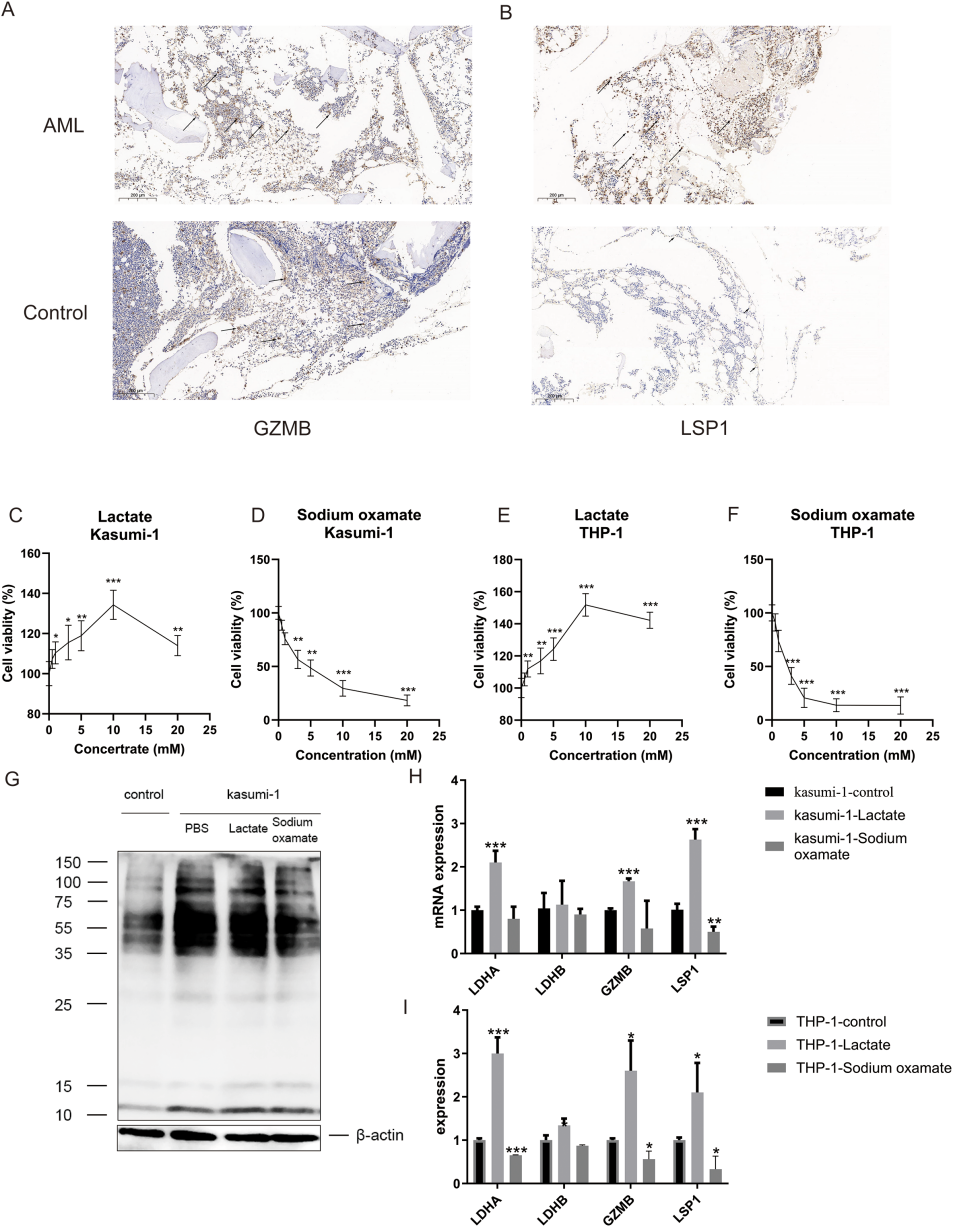
The expression of GZMB and LSP1 in AML patients is influenced by the lactate concentration. **(A, B)** Expression of GZMB **(A)** and LSP1 **(B)** in AML patients and healthy donors (n=3), as determined by IHC. **(C-F)** Viability of Kasumi-1 **(C, D)** and THP-1 **(E, F)** cells after treatment with lactate **(C, E)** and sodium oxamate **(D, F)**, as determined using a CCK-8 assay. **(G)** Pan-lactylated protein levels in Kasumi-1 cells and healthy donors (control), as determined by WB. **(H, I)** Expression of the LDHA, LDHB, GZMB, and LSP1 mRNAs in Kasumi-1 **(H)** and THP-1 **(I)** cells after treatment with lactate and sodium oxamate, as measured by qRT–PCR. *p value < 0.05, **p value < 0.01, ***p value < 0.001.

IHC was conducted on samples from AML patients and healthy donors to determine the expression levels of the GZMB and LSP1 proteins, and the results revealed higher expression in AML (**Figures 10A, B**). Given our previous findings concerning the effects of lactate on AML cells and the expression of GZMB and LSP1, we performed a CCK-8 assay to investigate the effects of lactate and its inhibitor, sodium oxamate, on cell viability and GZMB and LSP1 mRNA expression in the AML cell lines Kasumi-1 and THP-1. The results demonstrated that low to moderate concentrations of lactate (<10 mM) promoted AML cell proliferation, whereas high concentrations had inhibitory effects that were potentially linked to changes in pH (**Figure 10C**). In contrast, sodium oxamate treatment uniformly reduced AML cell viability (**Figure 10D**). Pan-lactylated protein levels were higher in Kasumi-1 cells than in cells from healthy donors, which is consistent with the results of the ssGSEA algorithm-based lactylation scoring system showing elevated lactylation activity in AML samples compared with that in controls. Lactate treatment further increased the pan-lactylated protein levels (**Figure 10E**) and increased the expression of the LDHA, GZMB, and LSP1 mRNAs, whereas LDHB expression remained unaffected. Sodium oxamate treatment reversed these changes (**Figures 10F, G**). These findings suggest a targeted regulatory relationship between lactate and GZMB/LSP1, with LDHA likely serving as a key enzymatic mediator.

### 3.11 Molecular docking indicates that (-)-gallocatechin gallate, indomethacin benzo(a)pyrene and benzo(e)pyrene might modulate key genes in AML

**Figure 11:**
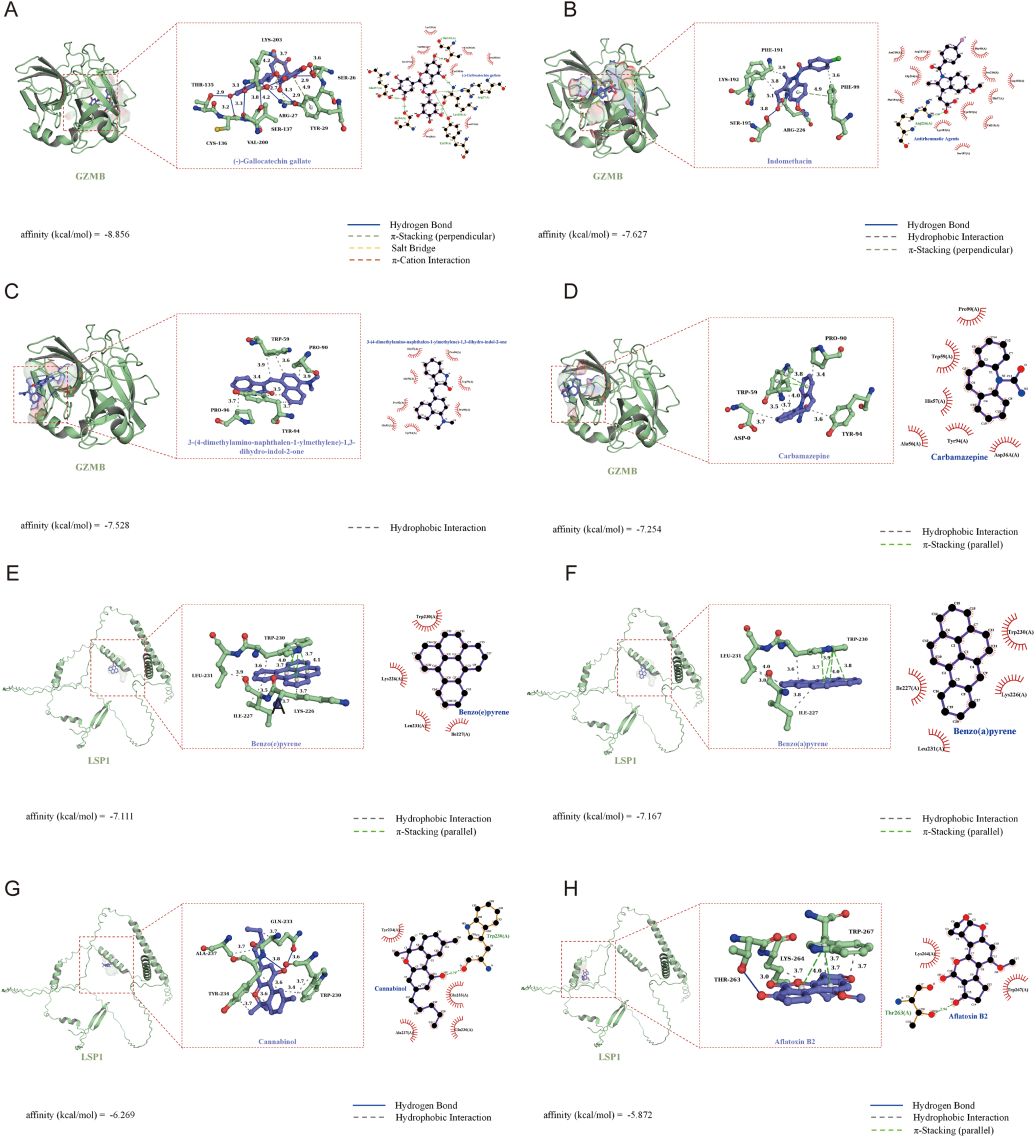
Molecular docking of GZMB and LSP1. **(A-D)** GZMB binding to (-)-gallocatechin gallate **(A)**, indomethacin **(B)**, 3-(4-dimethylamino-naphthalen-1-ylmethylene)-1,3-dihydro-indol-2-one **(C)**, and carbamazepine **(D)**. **(E-H)** LSP1 binding to benzo(a)pyrene **(E)**, benzo(e)pyrene **(F)**, cannabinol **(G)**, and aflatoxin B2 **(H)**.

As described previously, information on the relationships between the GZMB and LSP1 genes and small-molecule drugs was obtained. The top 10 drugs targeting GZMB and LSP1 were subsequently selected for target protein–ligand docking (**Figure 11**). The docking results showed that the binding energies of both interactions were less than -1.2 kcal/mol, indicating good binding effects. These values indicate favorable binding interactions, suggesting that all drugs could modulate GZMB or LSP1 activity or disrupt their functions in AML cells. However, not all binding interactions suppress protein expression. According to the literature, indomethacin, dronabinol, and dichlorvos inhibit GZMB expression or secretion, whereas LSP1-associated drugs have not been reported to suppress its expression. Further experimental validation may be needed to confirm these observations (**Supplementary Table 8**).

## 4 Discussion

In the treatment of acute myeloid leukemia (AML) and research on resistance mechanisms, the critical role of the bone marrow microenvironment in disease progression has been increasingly recognized (39). There have been models of the AML microenvironment, but they lack characteristic markers (40, 41). The Warburg effect reveals a pattern in which AML cells tend to perform their metabolic functions through glycolysis, resulting in increased lactate production within the BMME. Recent studies have highlighted the role of this metabolic reprogramming, such as STAT5-mediated lactylation, in modeling immune suppression in AML pathogenesis (42, 43). In this study, we found that this metabolic reprogramming correlated with AML immune responses and played vital roles in leukemia development and resistance. By integrating single-cell sequencing and transcriptome sequencing from public databases, and employing various bioinformatics and molecular biology approaches, we have mapped the lactate-lactylation-LRGs network in AML. Overall, our findings indicated that GZMB and LSP1 might be not only markers of a poor prognosis for AML patients but also potential therapeutic targets. Indomethacin is a commonly used drug in clinical practice, and its regulation of genes such as GZMB and its impact on the immune system still need to be carefully explored.

Previous studies have also confirmed that lactate accumulation in the peripheral blood and bone marrow of AML patients is associated with disease progression (9, 42). At present, there have been a few discussions on lactate-related models in AML, but they mainly focus on the lactic acid metabolic process, such as models like hexokinase-1 (HK1) (17, 20). Additionally, there are AML microenvironment-related models that aid in prognosis analysis, but the lack of quantifiable standards makes clinical implementation challenging (40, 41). This study comprehensively delineates the relationship between lactate-lactylation-LRGs and the microenvironment, which distinguishes it from previous research. While demonstrating potential for clinical translation, further validation in large-scale patient cohorts remains necessary.

The seven genes preliminarily identified in this study can be roughly divided into immune/inflammatory-related genes, namely, HLA-DRA, HLA-DRB1 (MHC class II complex components) and GZMB (T and NK cells), and stemness/proliferation-related genes, namely, SPINK2, LSP1, MPO and POU2F2. Granzyme B (GZMB) is a key effector molecule of cytotoxic T cells and natural killer cells (NK cells) that induces the apoptosis of target cells by activating the caspase pathway and participates in antitumor and anti-infection immune responses. In this study, GZMB was highly expressed in AML, especially in NK and DC cells, suggesting that GZMB activates antitumor immunity in AML (44). However, GSEA of HALLMARKS revealed the possibility that GZMB regulates pathways related to allograft rejection and subsequently leads to treatment failure, which is meaningful for further verification and study. LSP1, SPINK2, MPO and POU2F2 participate in maintaining the stemness of LSCs in AML. LSP1 (lymphocyte-specific protein 1) is an F-actin-binding protein that is expressed mainly in lymphocytes, neutrophils, and endothelial cells and, in this study, it was expressed mainly in pDCs. It is involved in cell migration, adhesion and phagocytosis (such as the phagocytic activity of macrophages) by regulating the dynamics of the actin cytoskeleton (45). In AML, LSP1 promotes the apoptosis of CD8^+^ T cells and increases AML resistance to sorafenib (46). Further mechanistic investigations into the role of LSP1 in AML are warranted.

Despite our best efforts, we acknowledge that our study has several limitations. While our multiomics approach provided comprehensive insights, several methodological limitations warrant consideration. The components of the bone marrow microenvironment are complex, but endothelial cells, osteoblasts, and Schwann cells were not analyzed. The single-cell data captured major hematopoietic lineages but underrepresented rare populations such as endothelial cells and osteoblasts. Hypoxia and lactylation have been shown to be associated with angiogenesis (47). In addition, the molecular components of the BMME, such as cytokines and chemokines, have not been discussed in depth. Lactate metabolism is an important type of metabolic reprogramming in tumors, which is related not only to the lactylation modification assessed in this study but also to metabolism in the body. The lactylation gene set was derived from the published literature, potentially omitting novel lactylation targets. Future studies could benefit from direct lactylation site detection through mass spectrometry and spatial transcriptomics to elucidate the microenvironmental organization. We will combine metabolomics and other methods to simulate the BMME *in vivo* and *in vitro* to verify our conclusions. The roles of lactate levels, lactylation marker detection and histone lactylation site detection in AML will be further explored, and interventions involving metabolic inhibitors and epigenetic regulatory drugs will be developed. Nevertheless, the consistent findings across multiple analytical platforms and validation cohorts support the robustness of our conclusions regarding the role of lactylation in AML pathophysiology. Our model demonstrated excellent predictive performance in the training set, but its performance declined when extended to the validation set. This indicates that further validation and transformation are necessary in a larger patient cohort in the future.

## 5 Conclusions

Through multiomics integration, this study established protein lactylation as a critical regulator of AML. We identified seven lactylation-related hub genes (LSP1, MPO, GZMB, SPINK2, HLA-DRB1, HLA-DRA, and POU2F2), among which GZMB and LSP1 exhibited a causal relationship with AML risk via Mendelian randomization. Lactate accumulation in the leukemic microenvironment promoted global protein lactylation, increasing AML cell proliferation —effects that were reversed by the lactate inhibitor sodium oxamate. A machine learning model incorporating these genes achieved robust prognostic predictions, while molecular docking highlighted indomethacin and (-)-gallocatechin gallate as potential therapeutic agents targeting GZMB/LSP1. These findings underscore lactylation-driven signatures as novel biomarkers and therapeutic targets, warranting further exploration of the modulation of lactate metabolism in AML treatment strategies.

## Data Availability

The datasets presented in this study can be found in online repositories. The names of the repository/repositories and accession number(s) can be found in the article/**Supplementary Material**.

## Glossary

AML: acute myeloid leukemia
ANOVA: one-way analysis of variance
AUC: area under the curve
BP: Biological Process
BMME: bone marrow microenvironment
CC: cellular component
DCA: decision curve analysis
DEGs: differentially expressed genes
EMBL-EBI: European Bioinformatics Institute
eQTL: expression quantitative trait loci
GO: Gene Ontology
GSEA: gene set enrichment analysis
GLM: generalized linear model
GTEx: Genotype–Tissue Expression
GMP: granulocyte–monocyte progenitor cells
GZMB: granzyme B
HLA: human leukocyte antigen
HSCT: hematopoietic stem cell transplantation
HSCs: hematopoietic stem cells
HOCl: hypochlorous acid
IVs: instrumental variables
KM: Kaplan–Meier
LD: linkage disequilibrium
LRGs: lactylation-related genes
LSCs: leukemic stem cells
LSP1: lymphocyte-specific protein 1
MAD: median absolute deviation
MEP: megakaryocyte–erythroid progenitor cells
MF: molecular function
MHC: major histocompatibility complex
MPO: myeloperoxidase
MR: Mendelian randomization
NK: cellsnatural killer cells
pDC: plasmacytoid dendritic cells
POU2F2: POU-like homeobox 2
PPI: protein–protein interaction
QC: quality control
RF: random forest
RF: random forest
ROS: reactive oxygen species
qRT−PCR: real‐time quantitative polymerase chain reaction
ROC: receiver operating characteristic curve
SNPs: single-nucleotide polymorphisms
SMZ: sulfamethoxazole
SPINK2: serine protease inhibitor Kazal-type 2
SVM: support vector machine
TCGA: The Cancer Genome Atlas
TMP-SMX: Compound sulfamethoxazole
UMAP: uniform manifold approximation and projection
WGCNA: eighted gene coexpression network analysis
XGB: extreme gradient boosting
