# Supplementary figures and images for: Integrative profiling of lactylation reveals prognostic biomarkers and an immunosuppressive niche in acute myeloid leukemia
Source: Front Immunol. 2026 Mar 23;17:1765979. doi: 10.3389/fimmu.2026.1765979 (PMC13050951; doi:10.3389/fimmu.2026.1765979)

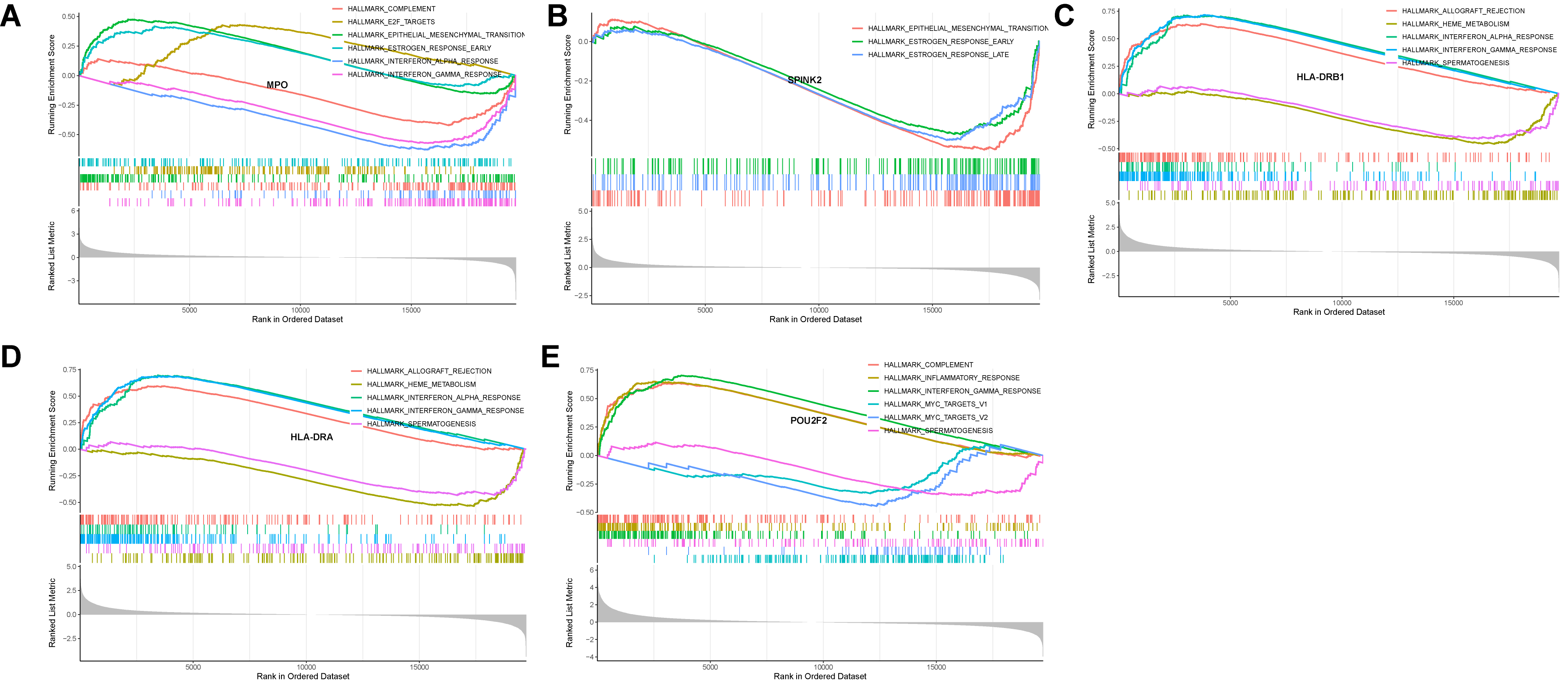

Supplement: Supplementary file 1 [file Image1.tif]

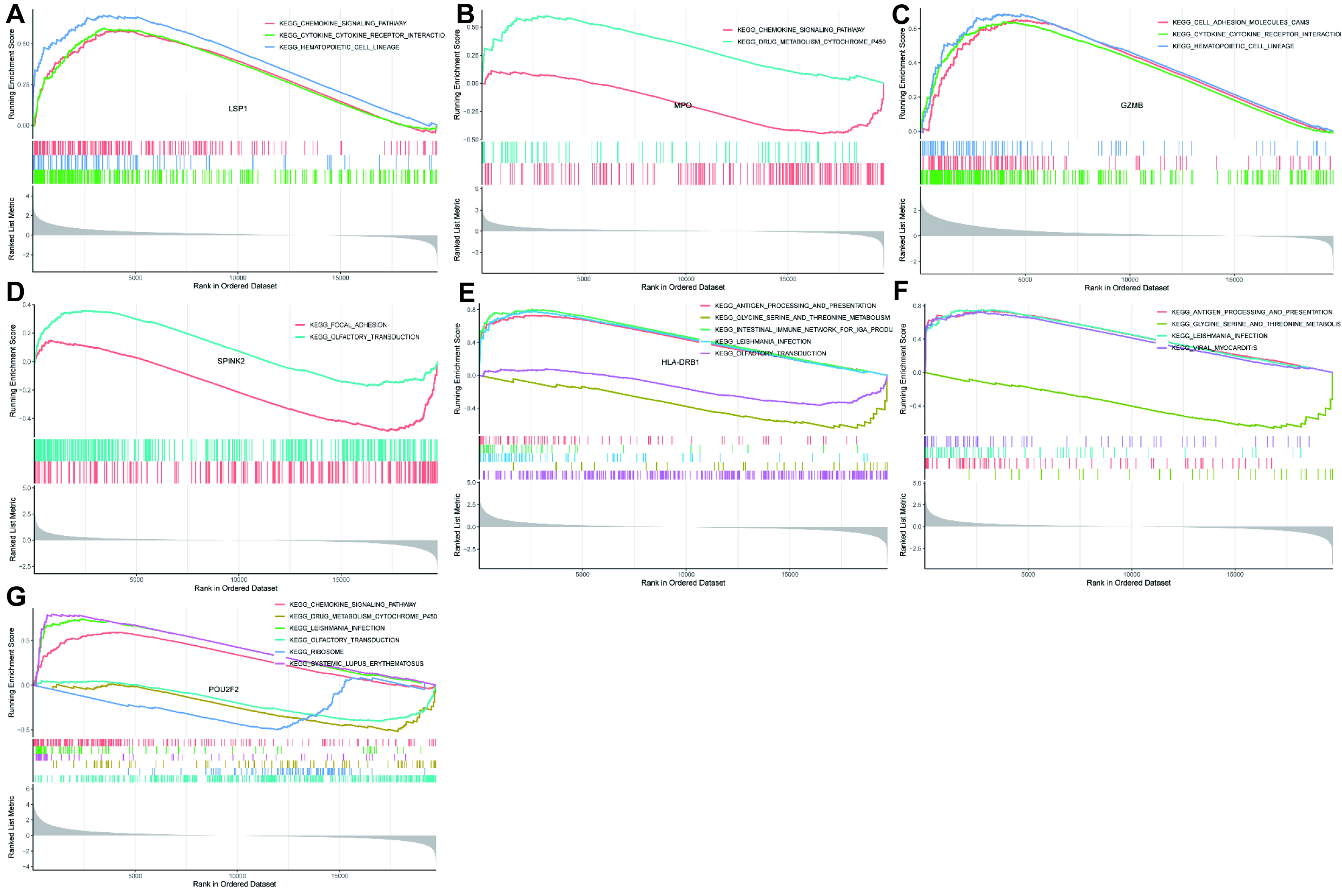

Supplement: Supplementary file 2 [file Image2.tif]

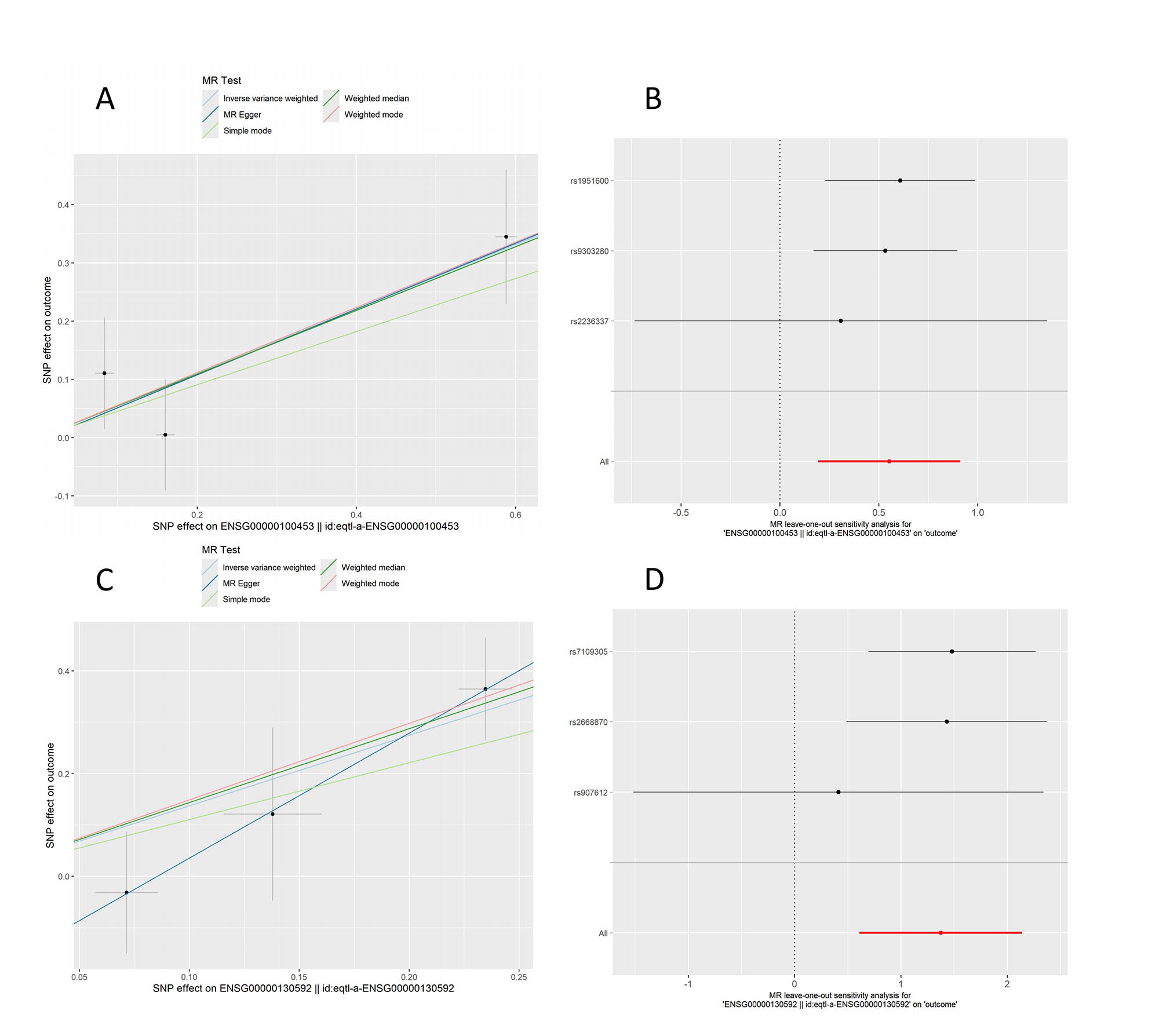

Supplement: Supplementary file 3 [file Image3.tif]
